# *Rictor*/TORC2 Regulates Caenorhabditis elegans Fat Storage, Body Size, and Development through *sgk-1*

**DOI:** 10.1371/journal.pbio.1000060

**Published:** 2009-03-03

**Authors:** Kevin T Jones, Elisabeth R Greer, David Pearce, Kaveh Ashrafi

**Affiliations:** 1 Department of Physiology and Diabetes Center, University of California San Francisco, San Francisco, California, United States of America; 2 Department of Medicine, University of California San Francisco, San Francisco, California, United States of America; Salk Institute for Biological Studies, United States of America

## Abstract

The target of rapamycin (TOR) kinase coordinately regulates fundamental metabolic and cellular processes to support growth, proliferation, survival, and differentiation, and consequently it has been proposed as a therapeutic target for the treatment of cancer, metabolic disease, and aging. The TOR kinase is found in two biochemically and functionally distinct complexes, termed TORC1 and TORC2. Aided by the compound rapamycin, which specifically inhibits TORC1, the role of TORC1 in regulating translation and cellular growth has been extensively studied. The physiological roles of TORC2 have remained largely elusive due to the lack of pharmacological inhibitors and its genetic lethality in mammals. Among potential targets of TORC2, the pro-survival kinase AKT has garnered much attention. Within the context of intact animals, however, the physiological consequences of phosphorylation of AKT by TORC2 remain poorly understood. Here we describe viable loss-of-function mutants in the Caenorhabditis elegans homolog of the TORC2-specific component, *Rictor* (*CeRictor*). These mutants display a mild developmental delay and decreased body size, but have increased lipid storage. These functions of *CeRictor* are not mediated through the regulation of AKT kinases or their major downstream target, the insulin-regulated FOXO transcription factor DAF-16. We found that loss of *sgk-1*, a homolog of the serum- and glucocorticoid-induced kinase, mimics the developmental, growth, and metabolic phenotypes of *CeRictor* mutants, while a novel, gain-of-function mutation in *sgk-1* suppresses these phenotypes, indicating that SGK-1 is a mediator of *CeRictor* activity. These findings identify new physiological roles for TORC2, mediated by SGK, in regulation of C. elegans lipid accumulation and growth, and they challenge the notion that AKT is the primary effector of TORC2 function.

## Author Summary

The target of rapamycin (TOR) kinase acts as a conserved sensor of energy status and governs diverse functions such as metabolism, growth, and cell size via two separate multiprotein complexes. TOR complex 1 (TORC1), which is sensitive to the immunosuppressant drug rapamycin, is well understood but the physiological roles and molecular mechanisms of action of the second TOR complex (TORC2) are not so clear. We describe mutants in the single Caenorhabditis elegans homolog of the gene *Rictor*, which is the defining component of the TORC2 signaling complex. Mutant worms are small, developmentally delayed, have reduced fecundity, and store more fat than wild-type C. elegans does. Akt kinases, which are pro-survival kinases that mediate the effects of insulin and other growth factors, have been postulated to be key mediators of TORC2 signaling, as they are targets of TORC2 phosphorylation. We find, however, that in C. elegans, TORC2 regulates fat storage, size, and development entirely independent of the Akt kinases and of the major target of insulin signaling, the FOXO-family transcription factor DAF-16. Instead, we show genetically that TORC2 acts through the activation of SGK-1, a kinase closely related to Akt, to govern all three phenotypes. This work indicates a role for TORC2 in fat regulation and shows that SGK-1 is a physiologically significant mediator of TORC2 signaling.

## Abstract 3

*C. elegans* TOR complex 2 regulates lipid storage, body size, and development through downstream activation of the SGK-1 kinase, independent of AKT kinases and of the DAF-16/FOXO transcription factor.

## Introduction

Target of rapamycin (TOR), a serine/threonine kinase of the phosphatidylinositol kinase-related family, is broadly conserved in eukaryotes, and in all systems examined, it is required for normal growth [1]. The TOR kinase acts as a sensitive cellular fuel gauge that receives inputs from multiple growth-promoting signals and, in turn, orchestrates a vast array of cellular responses such as translational control, ribosome biogenesis, and autophagy [1]. TOR's control of these vital cellular processes makes it, on the organismal scale, a critical player in numerous disease states, such as cancer, aging, and cardiovascular disease [2].

One reason for the immense diversity of TOR-regulated processes is that the kinase operates in two complexes, each with its own unique set of components and subsequent substrates [3]. The proteins Raptor and Rictor (rapamycin-insensitive companion of mTOR) are mutually-exclusive binding partners for TOR and define the TORC1 and TORC2 complexes, respectively [3]. TORC1 can also be distinguished from TORC2 functionally, because only TORC1 is inhibited by the compound rapamycin [3–5]. The use of this drug has been the major driving force in the study of the TOR kinase—it enabled the identification of key TORC1 targets [6–8], and the TOR kinase itself was first discovered by genetic analysis in yeast aimed at determining the mechanism of rapamycin's growth-inhibitory action [9].

Deciphering physiological functions of TORC2 has been hampered by lack of pharmacological inhibitors and lethality of yeast and rodent *Rictor* mutants, although the development of tissue-specific *Rictor* and *Raptor* knockout mice has recently been reported [10–12]. While *TOR-* and *Raptor*-deficient mouse embryos die soon after implantation, *Rictor*-deficient embryos die in midgestation around embryonic day 10.5 (E10.5), suggesting that TORC2 is not required for early proliferative and developmental events. *Rictor* knockout mice are, at the time of embryonic arrest, slightly smaller and developmentally delayed compared to wild-type littermates [13,14]. The first viable *Rictor* mutant to be studied in any organism was *Pianissimo* (*Pia*), the single *Dictyostelium* homolog. These mutants are unable to activate adenylyl cyclase in response to chemotactic GPCR signaling [15]. Viable *Rictor* mutants have also recently been described in *Drosophila* [16,17]. Consistent with the mouse data, reported phenotypes of *Drosophila Rictor* mutants include a mild developmental delay and an overall reduction in body size [16,17].

Another source of complexity in understanding the physiological roles of TORC2 is that, like any kinase, it has multiple substrates. A seminal discovery in elucidating the functions of TORC2 was its identification as a kinase activator of AKT [18], which is a key mediator of signaling of insulin and other pro-growth factor pathways and a kinase that is inappropriately activated in numerous cancers [19]. AKT, like all related AGC family kinases, is phosphorylated at several distinct sites, including the activation loop, mediated by PDK1, and the C-terminal turn and hydrophobic motifs, mediated by TORC2 [18,20,21]. Although hydrophobic motif (HM) phosphorylation of AKT has become a major readout for TORC2 function, it is not required for AKT to phosphorylate many of its substrates [22]. As in mammals, phosphorylation of the HM domain of *Drosophila* AKT is severely reduced in *Rictor*-deficient flies [16,17], yet AKT functions remain largely intact [16]. Similarly, removal of this phosphorylation site from the *Drosophila* AKT does not prevent its capacity to restore normal growth to tissues lacking AKT [16]. Thus, while HM motif phosphorylation of AKT increases its kinase activity in vitro [23] and has been postulated to permit maximal levels of kinase activity in vivo [16], the physiological significance of AKT as an effector of TORC2 in intact animals has been difficult to assess. Other AGC family kinases, such as PKC and SGK (serum- and glucocorticoid-regulated kinase), contain the conserved HM motif and have been proposed to mediate in vivo functions of TORC2 in yeast [24,25], yet the interplay of TORC2 and its various potential effectors in animals remain poorly understood.

Another complicating factor in elucidating the physiological functions of TORC2 is that genetic analyses of TORC2 in mammalian cell culture and in intact animals have yielded differing results. For instance, siRNA-induced knockdown of *Rictor* in mammalian cells disrupts the actin cytoskeleton [26], but ablation of *Rictor* through homologous recombination causes lethality without notable effects on the actin cytoskeleton [13,14]. Together, these observations raise the need for disentangling the TOR signaling network within the context of intact animals, where contributions of various potential effectors of TORC2 on size, growth, and other potential physiological processes can be rigorously examined.

Here, we report the identification of viable, loss-of-function *Rictor* mutants in C. elegans. In addition to these mutants having body size and growth defects, similar to previous reports of *Rictor* mutants in other organisms [16], we demonstrate that they also show an increase in stored fat. Our genetic analyses of these mutants indicate that TORC2 regulates the observed fat storage, size, and developmental phenotypes through *sgk-1*, rather than the AKTs. These findings reveal that the SGK kinase family is a key physiological mediator of the pro-growth and lipid storage effects of TORC2.

## Results

### Identification of *C. elegans Rictor* Mutants

**Figure 1:**
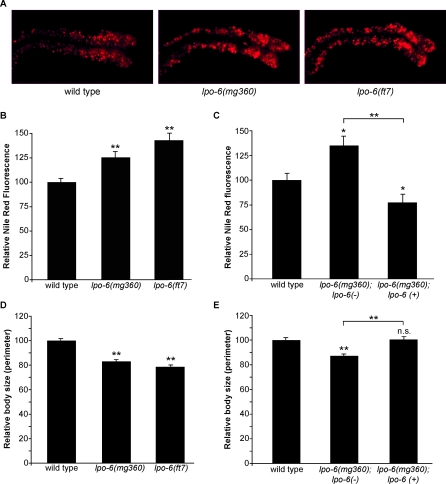
Loss of *CeRictor* Causes Increased Lipid Storage and Decreased Body Size (A) Images of Nile Red staining of lipids in anterior intestinal cells of adult wild-type, *lpo-6* (*mg360*), and *lpo-6* (*ft7*) strains. Representative images are shown. In each image, anterior of the animal is to the right. (B) Quantification of Nile Red staining. Mean fluorescence intensity is reported as a percentage of the mean for wild-type animals (error bars indicate s.e.m.). Single asterisk indicates a *p*-value < 0.05, and double asterisk indicates *p*-value <0.01 (wild type versus mutant, two-tailed *t*-test, *n* = 5–7). (C) Quantification of Nile Red staining. Mean fluorescence intensity is reported as a percentage of the mean for wild-type animals (error bars indicate s.e.m.). Single asterisk indicates a *p*-value ≤ 0.05, and double asterisk indicates p-value < 0.01 (wild type versus mutant, two-tailed *t*-test, *n* = 5–8). *lpo-6* (*+*) and (*-*) refer to the presence or absence of wild-type *CeRictor* transgene. (D) Quantification of body size measurements. The perimeter of each worm was measured. Values reported as mean size as a percentage of mean for wild type (error bars indicate s.e.m.). Double asterisk indicates *p*-value < 0.01 (wild type versus mutant strain, two-tailed *t*-test, *n* = 10). (E) Quantification of body size measurements. The perimeter of each worm was measured. Values reported as mean size as a percentage of mean for wild type (error bars indicate s.e.m.). Double asterisk indicates *p*-value < 0.01 (wild type versus mutant strain, two-tailed *t*-test, *n* = 10). *lpo-6* (*+*) and (*-*) refer to the presence or absence of wild-type *CeRictor* transgene. Body size of rescued *lpo-6* mutants (*lpo-6* (*mg360*)*; lpo-6* (*+*)) is not significantly different from wild-type animals (*p*-value = 0.88, two-tailed *t*-test, *n* = 10).

In a screen aimed at identifying C. elegans mutants with altered lipid storage, we isolated *lpo-6* (*mg360*), an allele of the single C. elegans homolog of TORC2-specific component *Rictor* (encoded by the gene *F29C12.3*). C. elegans stores fat in intestinal and skin-like hypodermal cells, and intestinal stores are readily detectable using the vital dye Nile Red [27]. Mutants identified in this screen were termed “*lpo*,” or lipophilic dye abnormal. The *lpo-6*(*mg360*) mutation results in a single amino acid substitution at a highly-conserved residue in the C-terminal region of the protein, which has been shown to be required for *Rictor* function in Saccharomyces cerevisiae [28] (Figure S1). The excess fat accumulation of *lpo-6* (*mg360*) mutants was associated with enlarged lipid-storing subcellular particles throughout intestinal cells, as well as an overall increase in Nile Red fluorescence (Figure 1A and 1B). We confirmed increased lipid storage phenotype of *lpo-6* (*mg360*) by staining with other lipid dyes—fatty acid-conjugated BODIPY [27,29] and the fixed stain Sudan Black [30] (Figure S2A and S2B). Overexpression of the wild-type, genomic *CeRictor* locus in *lpo-6* (*mg360*) mutants not only reversed the increased fat phenotype of this mutant but resulted in lower-than-wild-type fat levels (Figure 1C), suggesting that the recessive *mg360* mutation is a loss-of-function allele. In addition to having excess fat, *lpo-6* mutants displayed decreased adult body size (Figure 1D) and a slight developmental delay, phenotypes quite similar to those noted in *Drosophila Rictor* mutants [16]. The decreased size and developmental delay of *lpo-6* (*mg360*) mutants were also rescued to wild type by transgenic expression of the wild-type genomic *CeRictor* locus (Figure 1E and unpublished data).

In an unrelated Nile Red screen, we later isolated another allele of *CeRictor*, *ft7*, which is likely to be a null, as this mutation results in a very early stop (Figure S1). Consistent with this interpretation, the *lpo-6* (*ft7*) mutation causes a greater increase in fat storage, smaller body size, and a more severe developmental delay than the *lpo-6* (*mg360*) mutation (Figure 1A, 1B, and 1D).

**Figure 2:**
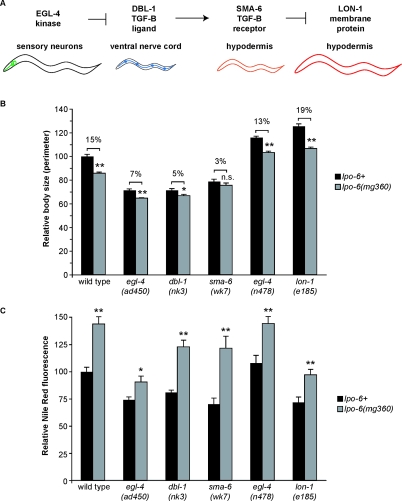
*CeRictor* Regulates Size Independent of the *dbl-1* Pathway (A) Genetic regulatory hierarchy of the *dbl-1*-mediated body size pathway. Molecular function, expression pattern, and representation of null phenotype for each component are indicated. (B) Quantification of body size measurements. The perimeter of each worm was measured. Sizes are reported as percentage of wild type (error bars indicate s.e.m). For each gene, sizes of both mutant strain alone (black bars) and mutant strain also harboring the *lpo-6* (*mg360*) mutation (gray bars) are shown. Asterisks indicate significance of difference between single mutant and *lpo-6* (*mg360*);mutant strain; single asterisk indicates *p*-value < 0.05 and double asterisk indicates *p*-value < 0.01 (two-tailed *t*-test, *n* = 10–20 per strain). *sma-6* (*wk7*) and *sma-6* (*wk7*)*;lpo-6* (*mg360*) are not significantly different (two-tailed *t*-test, *p* = 0.24). Percentages above bars represent the percent reduction in *lpo-6* (*mg360*); mutant strain as compared to mutant strain alone (percentage refers to size as a percentage of wild type). (C) Quantification of Nile Red staining. Mean fluorescence intensity is reported as a percentage of the mean for wild-type animals (error bars indicate s.e.m.). For each gene, sizes of both mutant strain alone (black bars) and mutant strain also harboring the *lpo-6* (*mg360*) mutation (gray bars) are shown. Asterisks indicate significance of difference between single mutant and *lpo-6* (*mg360*); mutant strain; single asterisk indicates *p*-value < 0.05, and double asterisk indicates *p*-value < 0.01 (two-tailed *t*-test, *n* = 5–7 per strain). As indicated in Materials and Methods, the reported values for fat correspond to Nile Red fluorescence within the first three pairs of intestinal cells without adjustment for differences in cell size between mutants.

To compare lipid staining at matched developmental stages, we allowed *CeRictor* mutants to grow for a longer period of time than wild-type animals (see Materials and Methods for details) so that we always assayed fat content at the start of egg-laying in all strains. The increased fat staining of *CeRictor* mutants was not the result of this correction, as animals grown for the same duration of time as wild type still displayed increased fat staining even though they were less mature (Figure S2C). The excess fat of *CeRictor* mutants was not simply a reflection of the same total fat contained within a smaller volume either. In fact, other similarly small-sized mutants did not display enhanced fat accumulation (Figure 2C). Together, these findings indicate that *CeRictor* functions to regulate fat storage, growth, and development.

### *CeRictor* Mutant Phenotypes Are Mimicked by Loss of Another TORC2 Component

While originally designated as a lethal gene inactivation, the loss of *CeTOR* kinase encoded by *let-*363 allows for completion of embryonic development but causes a nonconditional larval-stage arrest with dauer-like phenotypes [31,32]*.* Loss of *daf-15/CeRaptor* also results in dauer-like developmental arrest [31,32]. By contrast, under identical growth conditions, *CeRictor* mutants and wild-type animals grow to reproductive adults without entry into the dauer stage. We found that animals in which the C. elegans homolog of *lst-8* (encoded by *C10H11.8*) was targeted by RNAi also grew to reproductive adults without dauer entry and, as adults, displayed the fat, size, and developmental delay phenotypes of *CeRictor* mutants (Figure S3 and unpublished data). *lst-8* encodes for a G protein β subunit–like protein whose mammalian homolog is found in both TORC1 and TORC2 complexes. Surprisingly, however, *mLST-8* mutant mice display the phenotypes of *Rictor*, not *Raptor*, mutants, and they are not defective in the phosphorylation of known TORC1 targets [14]. Thus, our findings are consistent with murine characterizations showing that *Rictor* mutant phenotypes are recapitulated by inactivation of *lst-8* while *TOR* mutant phenotypes are recapitulated by *Raptor* inactivation. While we are unable to rule out the possibility that RNAi may not fully abrogate *lst-8* expression, these data support the notion that phenotypes associated with *lpo-6* mutations are due to reduced TORC2 signaling.

### Expression Pattern of *CeRictor*

To determine the spatial relationship between *Rictor* and other components of TOR complex, we examined the expression pattern of a GFP reporter fusion controlled by the rescuing promoter of *CeRictor*. GFP reporter fusions to *let-363/CeTOR* and *daf-15/CeRaptor* are expressed throughout development and adulthood in most tissues, including intestine, skin-like hypodermis, pharyngeal and body wall muscle, and the nervous system [31,32]. Expression of *CeRictor* reporter fusion was limited to the intestinal and hypodermal cells at early larval stages. As animals grew to the adult stage, visible expression was restricted to the intestinal cells and a single pharyngeal interneuron, I1 (Figure S1C–S1E). We do not yet know the relevance of the observed I1 expression; however, expression of the GFP reporter fusion in key fat-storing tissues of C. elegans is consistent with a role for *CeRictor* in regulating fat accumulation.

### *CeRictor* Acts Independently of the DBL-1 TGF-β Pathway to Regulate Body Size

Because *Rictor* mutants in both the fly and mouse display a proportionate and subtle decrease in body size [13,16], we chose to examine the mechanisms by which *CeRictor* regulates size. Changes in either cell number or cell size may alter total body size. The reduced body size of *lpo-6* mutants, however, is unlikely to be the result of a failure in cell proliferation, as there are no obvious groups of cells missing or reduced in number in *lpo-6* mutants. These animals only show a size defect after they complete larval development (unpublished data). Together with the fact that the C. elegans cell lineage is highly invariant, these findings suggest that the small size of *CeRictor* mutant reflects a defect in achieving post-mitotic cell size.

The major size-regulatory pathway in C. elegans is mediated by a TGF-β ligand, DBL-1, that connects signaling upstream in the nervous system to the skin-like hypodermal cells and modulates cell size throughout the organism in a dose-dependent manner [33]. Size regulatory *egl-4*, *dbl-1*, *sma-6*, and *lon-1* define a linear genetic pathway (Figure 2A). The EGL-4 cGMP-dependent protein kinase is required for mediating food-related sensory cues that elicit diverse behavioral and physiological responses [34]; it also acts in sensory neurons to regulate body size [35]. Gain-of-function mutations in *egl-4* [e.g., *egl-4* (*ad450*)], cause a decrease in size, whereas loss-of-function mutations [e.g., *egl-4* (*n478*)] result in increased size [35,36]. The large size of loss-of-function *egl-4* mutants is fully suppressed by loss of *dbl-1* [35]. The DBL-1 ligand is produced in the neurons of the nerve cord that extends the length of the animal, and it signals through the SMA-6 and DAF-4 TGF-β receptors acting in the hypodermis to promote normal size [37,38]. In turn, the SMA-6/DAF-4 receptors regulate the transcription of numerous genes that specifically influence cell growth; one such target is LON-1, a protein of uncharacterized biochemical function whose transcription is repressed by DBL-1 and SMA-6. Loss-of-function mutations in *lon-1* increase body size and suppress the small size of *dbl-1* and *sma-6* mutants [39,40].

We probed the relationship between *CeRictor* signaling and this previously-characterized size pathway by constructing double mutant strains bearing the *lpo-6* (*mg360*) mutation and other size-altering mutations. The *lpo-6* (*mg360*) mutation decreased body size in nearly all backgrounds examined (Figure 2B). Several observations indicated that *CeRictor* cannot be placed within a linear genetic relationship with other size-regulatory pathway components. First, the small-sized *lpo-6* (*mg360*) mutant was not fully suppressed by size-increasing *egl-4* and *lon-1* mutations, nor did it fully suppress the effects of these mutations (Figure 2B). Second, loss of *CeRictor* had a much greater effect in normal-sized or large strains, compared to small-sized strains (Figure 2B). For example, loss of *CeRictor* reduced the size of *lon-1* mutants by nearly 20%, whereas it reduced the size of *dbl-1* or *sma-6* mutants by only 3–5%, a 4- to 6-fold difference. Thus, rather than *CeRictor* acting as a component of the size-regulatory *dbl-1* pathway, these epistatic relationships indicate that *CeRictor* has a permissive function acting in parallel with the *dbl-1* pathway to shape the final organismal form. In support of this hypothesis, the *lpo-6* (*mg360*) mutation resulted in increased lipid staining in every size-altering background, suggesting that *CeRictor* does not act through the DBL-1 pathway (Figure 2C).

The excess fat storage and genetic relationship of *CeRictor* mutants with other size-pathway mutants suggested that *CeRictor* may influence organismal size by regulating the utilization of resources toward energetic processes like cell growth and development. As such, because of an increased demand for resources, mutants that display excessive cell size (such as the *lon-1* and *egl-4* loss-of-function mutants) would be more dependent upon growth-permissive TORC2 signaling than mutants with modest size.

### *CeRictor* Mutants Display Wild Type Feeding Behavior but Decreased Fecundity

**Figure 3:**
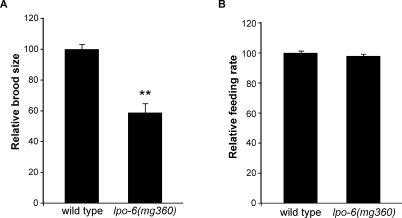
Inactivation of *CeRictor* Does Not Affect Feeding Rate but Is Required for Normal Reproduction (A) Total number of progeny was counted for individual wild-type or *lpo-6* (*mg360*) animals (*n* = 10 per strain). Average brood sizes are expressed as a percentage of wild type (error bars indicate s.e.m.). Double asterisk indicates *p*-value < 0.01(two-tailed *t*-test). (B) Feeding rate (pharyngeal pumping) was measured in *lpo-6* (*mg360*) and wild-type animals (*n* = 20 for each strain). Average rate is expressed as a percentage of wild type (error bars indicate s.e.m.). Strains were measured as well-fed adults. Wild-type and *lpo-6* (*mg360*) animals are not significantly different (*p* = 0.14, two-tailed *t*-test).

To further characterize the role of *CeRictor* in energy balance, we examined whether *CeRictor* activity is also necessary to promote other energetically demanding processes. *CeRictor* mutants had grossly normal rates and patterns of locomotion (unpublished data). By contrast, these animals produced ∼40% fewer progeny than wild type, resulting in over 100 fewer offspring on average (Figure 3A). We also examined food intake behavior of *CeRictor* mutants by measuring the pharyngeal pumping rate [41]. The more severe feeding-defective (*eat*) mutants share some phenotypes with *CeRictor* mutants—decreased size, slow growth, and reduced brood size [41]. Unlike the *eat* mutants, however, the pumping rate of *lpo-6* (*mg360*) animals was indistinguishable from that of wild-type animals under well-fed conditions (Figure 3B), suggesting that the increased lipid storage of *CeRictor* mutants is not attributable to feeding behavior and that their pleiotropic phenotypes are not simply due to insufficient nutrient intake at the level of feeding. Our findings suggest that TORC2 activity promotes body size, development, and progeny production in a regulated fashion, consistent with the known role of TOR kinase as a gauge of nutrient availability that coordinates utilization of energetic resources. Moreover, the severity of phenotypes caused by loss of *CeRaptor* relative to those caused by *CeRictor* indicate that the TOR kinase can fine tune responses of the organism to environmental conditions.

### *CeRictor* Regulation of Lipid Storage, Size, or Development Is Independent of AKT and DAF-16/FOXO Signaling

**Figure 4:**
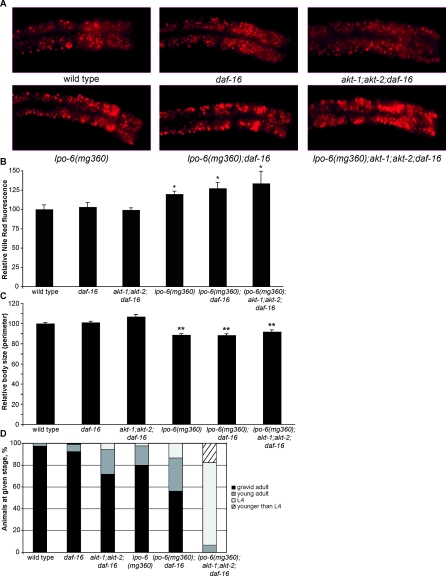
*CeRictor* Regulation of Lipid Storage, Size, and Development Is Independent of *Akt* and *FOXO* Function (A) Images of Nile Red staining of lipids in anterior intestinal cells. (B) Quantification of Nile Red staining. Mean fluorescence intensity is reported as a percentage of the mean for wild-type animals (error bars indicate s.e.m.). Asterisk indicates *p*-value < 0.05 (wild type versus mutant, two-tailed *t*-test, *n* = 4–5). *lpo-6* (*mg360*)*;daf-16* and *lpo-6* (*mg360*)*;akt-1;akt-2;daf-16* are not significantly different from *lpo-6* (*mg360*) (two-tailed *t*-tests, *p*-values for both comparisons are 0.44). (C) Quantification of body size. Values are reported as mean size as a percentage of mean for wild type (error bars indicate s.e.m.). Double asterisk indicates *p*-value < 0.01 (wild type versus mutant strain, two-tailed *t*-test, *n* = 10). *lpo-6* (*mg360*)*;daf-16* and *lpo-6* (*mg360*)*;akt-1;akt-2;daf-16* are not significantly different from *lpo-6* (*mg360*) (two-tailed *t*-test, *p*-values are 0.84 and 0.12, respectively). (D) Developmental timing of mutant strains. Animals were grown from synchronized L1s for 72 h at 20 °C; individuals were then scored for developmental stage. Fifty nine–156 animals were used for each strain.

To investigate the molecular mechanisms through which *CeRictor* regulates fat storage, size, and development, we first focused on *Akt* and insulin signaling. AKT phosphorylation at the HM site by TORC2 has been hypothesized to be a major output of TORC2 [18,22], and the *Akt*/insulin signaling pathway is a major regulator of metabolism and growth [42]. C. elegans has two AKT homologs that act redundantly to phosphorylate the FOXO transcription factor, DAF-16, which is a key effector of responses elicited upon reduced insulin signaling [43]. Inactivation of the *daf-2* insulin receptor or simultaneous inactivation of both *Akt* genes results in dauer arrest that is fully suppressed by mutation of *daf-16* [43]. If *CeRictor* acts solely through activation of the *Akt* genes to inhibit DAF-16/FOXO, then null mutations in *daf-16* should suppress all *CeRictor* mutant phenotypes. Strikingly, we found that elimination of *daf-16/FOXO* did not alter the lipid storage, body size, nor developmental phenotypes of *CeRictor* mutants (Figure 4A–4D). These results show that *CeRictor* acts in parallel to or downstream of *daf-16* in regulation of size, rate of development, and fat accumulation.

Next, we explored the possibility that *CeRictor* functions through the *Akt* pathway but independent of its DAF-16 output. To avoid dauer arrest caused by inactivation of both *Akt* genes and to specifically investigate *daf-16*-independent outputs of *Akt* signaling, we asked whether *akt-1; akt-2; daf-16* triple mutants mimicked any of the phenotypes seen in *CeRictor* mutants. These triple mutants accumulated nearly wild-type fat levels (Figure 4A and 4B), exhibited slightly elevated body size (Figure 4C), and displayed a very mild developmental delay (Figure 4D). Moreover, the quadruple *lpo-6* (*mg360*)*; akt-1; akt-2; daf-16* mutant strain was no different with respect to fat storage or body size than *lpo-6* (*mg360*) alone (Figure 4A–4C). These results show that the excess fat and reduced size of *CeRictor* mutant are unlikely to be consequences of reduced AKT activity, because complete removal of AKT activity does not mimic or alter these phenotypes. Furthermore, we found that the loss of *CeRictor* in the context of *akt-1*, *akt-2*, and *daf-16* mutations resulted in a synthetic reduction in developmental rate (Figure 4D), delaying the onset of egg-laying by nearly 24 h relative to wild type [compared to a roughly 6-h delay in *lpo-6* (*mg360*) alone]. This again suggests that the growth delay of *CeRictor* mutants is not merely a reflection of reduced AKT activity but that *CeRictor* acts in parallel to a novel, *daf-16–*independent *Akt* signaling pathway to regulate development. These results do not exclude the possibility that *CeRictor* may activate *Akt* for other physiological processes or under other conditions, but they strongly indicate that the fat, size, and developmental phenotypes of *CeRictor* are not consequences of compromised AKT signaling.

### *CeRictor* Acts in a Pathway with *sgk-1* to Regulate Lipid Storage, Size, and Development

**Figure 5:**
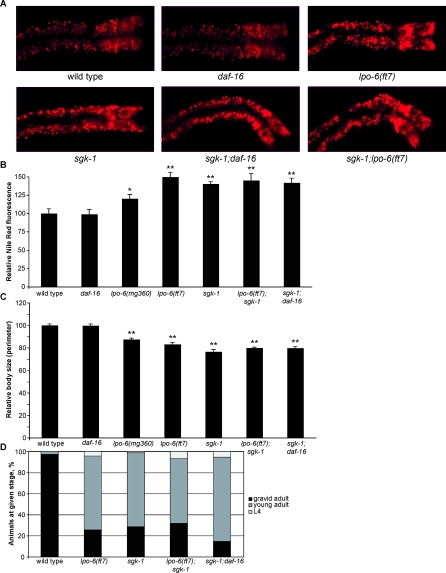
*CeRictor* and *sgk-1* Define a Single Regulatory Pathway Governing Fat Storage, Body Size, and Developmental Rate (A) Images of Nile Red staining of lipids in anterior intestinal cells. (B) Quantification of Nile Red staining. Mean fluorescence intensity is reported as a percentage of the mean for wild-type animals (error bars indicate s.e.m.). Single asterisk indicates *p*-value < 0.05, and double asterisk indicates *p*-value < 0.01 (wild type versus mutant, two-tailed *t*-test, *n* = 5–6). *lpo-6* (*ft7*) is not significantly different from *sgk-1* (two-tailed *t*-test, *p*-value = 0.20). *sgk-1* does not statistically differ from *sgk-1;lpo-6* (*ft7*) or *sgk-1;daf-16* (two-tailed *t*-tests, *p*-values = 0.63 and 0.81, respectively). (C) Quantification of body size. Values reported as mean size as a percentage of mean for wild type (error bars indicate s.e.m.). Double asterisk indicates *p*-value < 0.01 (wild type versus mutant strain, two-tailed *t*-test, *n* = 10). *sgk-1* does not statistically differ from *sgk-1;lpo-6* (*ft7*) or *sgk-1;daf-16* (two-tailed *t*-tests, *p*-values = 0.11 and 0.14, respectively). (D) Developmental timing of mutant strains. Animals were grown from synchronized L1s for 72 h at 20 °C; individuals were then scored for developmental stage. Sixty–185 animals were used for each strain.

Since the AKTs and DAF-16/FOXO were not the relevant outputs for *CeRictor* with respect fat, size, and developmental rate, we investigated the possibility of TORC2 regulation of these processes through other AGC family kinases. Knockdown of the three C. elegans PKC homologs resulted in visible phenotypes, but not those associated with *CeRictor* mutants (unpublished data). Strikingly, however, targeting the single SGK homolog, *sgk-1*, by RNAi resulted in fat, size, and developmental phenotypes strongly resembling *CeRictor* mutants (unpublished data). We confirmed these RNAi phenotypes by using an *sgk-1* mutant likely to be a null, because a deletion in this strain removes most of the region encoding the SGK-1 kinase domain [44]. *sgk-1* null mutants displayed a fat accumulation phenotype very similar to that of *lpo-6* (*ft7*) (the likely null allele of *CeRictor*), both in terms of enlarged lipid particle morphology (Figure 5A) and total fluorescence (Figure 5B). As previously reported [44], *sgk-1* mutants had decreased size and a slight developmental delay, and these phenotypes were highly similar to those of *lpo-6* (*ft7*) animals (Figure 5C and 5D).

**Figure 6:**
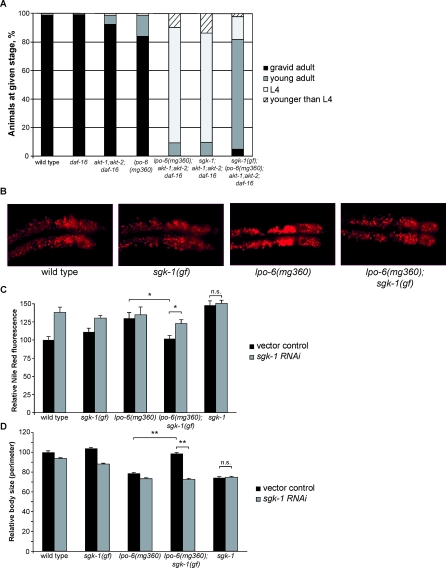
A Gain-of-Function Mutation in *sgk-1* Suppresses the Fat Storage, Body Size, and Developmental Phenotypes of *lpo-6* (*mg360*) (A) Developmental timing of mutant strains. Animals were grown from synchronized L1s for 72 h at 20 °C; individuals were then scored for developmental stage. Sixty–185 animals were used for each strain. One hundred and six–181 animals were used for each strain. In all panels of this figure, *sgk-1* (*gf*) refers to the described *sgk-1* (*ft15*) allele, and *sgk-1* refers to the null deletion allele, *sgk-1* (*ok538*). (B) Images of Nile Red staining of lipids in anterior intestinal cells. (C) Quantification of Nile Red staining. Mean fluorescence intensity is reported as a percentage of the mean for wild type or vector control (error bars indicate s.e.m.). Asterisk indicates *p*-value < 0.05 (*lpo-6* (*mg360*)*;sgk-1* (*gf*) grown on vector control versus *sgk-1* RNAi, or *lpo-6* (*mg360*) grown on vector control versus *lpo-6* (*mg360*)*;sgk-1* (*gf*) grown on vector control, two-tailed *t*-test, *n* = 5–6). *sgk-1* grown on vector control does not differ significantly from *sgk-1* grown on *sgk-1* RNAi (two-tailed *t*-test, *p*-value = 0.77). (D) Quantification of body size. Values reported as mean size as a percentage of mean for wild type on vector control (error bars indicate s.e.m.). Double asterisk indicates *p*-value < 0.01 (*lpo-6* (*mg360*)*;sgk-1* (*gf*) grown on vector control versus *sgk-1* RNAi, or *lpo-6* (*mg360*) grown on vector control versus *lpo-6* (*mg360*)*;sgk-1* (*gf*) grown on vector control, two-tailed *t*-test, *n* = 10). *sgk-1* grown on vector control does not differ significantly from *sgk-1* grown on *sgk-1* RNAi (two-tailed *t*-test, *p*-value = 0.89).

Both *CeRictor* and *sgk-1* are expressed in the intestinal cells, raising the possibility of a direct regulatory connection [44]. To ascertain whether *CeRictor* and *sgk-1* define a singular pathway, we examined the phenotypes of *lpo-6; sgk-1* double mutants. Although each null allele on its own had robust Nile Red, size, and developmental phenotypes, the double mutant showed no enhancement of any of the three phenotypes when compared with *sgk-1* alone (Figure 5A–5D), demonstrating that *lpo-6* and *sgk-1* act in the same pathway to regulate these processes. Consistent with *CeRictor's daf-16*–independent function, we found that the complete loss of *daf-16* did not suppress or otherwise alter the fat, size, and developmental delay phenotypes of *sgk-1* mutants (Figure 5A–5D). All previously described phenotypes of *sgk-1* inactivation in C. elegans (increased tendency for dauer formation when combined with other mutations, enhanced stress resistance, and extended lifespan) had been shown to operate entirely through the inhibition of DAF-16/FOXO [44]. Thus, our findings indicate that additional mediators of SGK function remain to be identified. Finally, *sgk-1* mutants also exhibited the same synthetic developmental delay phenotype in the context of the *akt-1; akt-2; daf-16* triple mutants (Figure 6A) that we observed with *lpo-6* (*mg360*) mutants (Figure 4D). Taken together, our genetic results indicate that *CeRictor* and *sgk-1* function in a shared pathway to regulate fat storage, size, and development. These results are consistent with findings in S. cerevisiae, where the TORC2 complex has been shown to directly phosphorylate the SGK homolog Ypk2 to regulate growth and ceramide synthesis [24,25].

### A Novel, Gain-of-Function Mutation in *sgk-1* Suppresses the Lipid Storage, Size, and Developmental Phenotypes of *CeRictor*

To demonstrate that *CeRictor* regulates lipid metabolism and growth by activating *sgk-1*, we first attempted to generate a constitutively active form of SGK-1. We reasoned that such an activated SGK-1 would rescue phenotypes caused by loss of *CeRictor*. We speculated that substitutions of glutamic acid or aspartic acid for the threonine at residue 444 in the HM domain of CeSGK-1, equivalent to S422 in mouse SGK-1, might result in a constitutively active SGK-1. We generated SGK-1 constructs with a glutamic acid substitution at Thr444 alone and aspartic acid substitutions simultaneously at Thr444 and the predicted phosphorylated residue within the turn motif. The introduction of these transgenes into *CeRictor* mutants did not, however, result in rescue of any of the phenotypes of these mutants (unpublished data). The structural and biochemical reasons why these amino acid substitutions failed to generate activated SGK-1 are not known, but these results are similar to what has been reported in S. cerevisiae. In that system, substituting aspartic acid residues at both the hydrophobic motif and turn motif phosphorylation sites on Ypk2 also fails to rescue *tor2* mutants [25].

Next, we undertook an unbiased, forward mutagenesis screen aimed at identifying suppressors of *CeRictor*. To isolate specific suppressors of *CeRictor*, we mutagenized *lpo-6* (*mg360*)*; akt-1; akt-2; daf-16* animals and screened the F_2_ generation for those that suppress this strain's developmental delay. As a secondary screen, we then searched for suppressors that also abrogate the increased Nile Red phenotype of the quadruple mutant strain. Reasoning that an activating mutation in *sgk-1* might suppress *CeRictor*, we sequenced the *sgk-1* locus in all potential mutants that passed both rounds of screening and identified one line with a mutation in *sgk-1*. This allele, *sgk-1* (*ft15*), has a G-to-A transition resulting in a Glu-to-Lys amino acid substitution at residue 116, shortly before the start of the kinase domain (Figure S4A and S4B). The *sgk-1* (*ft15*) mutation partially suppresses the synthetic developmental delay of the *lpo-6; akt-1; akt-2; daf-16* strain (Figure 6A), and exposure of *lpo-6; akt-1; akt-2; daf-16*; *sgk-1* (*ft15*) quintuple mutants to *sgk-1* RNAi abrogated the effects of the *ft15* allele, suggesting that this allele is likely a gain-of-function mutation in *sgk-1* (unpublished data)*.*

To determine its effect on *CeRictor* alone, we outcrossed the *sgk-1*(*ft15*) mutation, removing the *akt-1* and *daf-16* mutations in the background; however, because *sgk-1* is linked to the *akt-2* locus, the outcrossed *ft15* mutant strain also retained the *akt-2* deletion, which we have shown does not suppress any of the phenotypes of *lpo-6* (*mg360*) (Figure 4A–4D). *sgk-1* (*ft15*) mutants displayed wild-type body size and fat, and the mutation completely suppressed both the decreased body size and increased fat phenotypes of *lpo-6* (*mg360*) mutants (Figure 6B–6D). The *ft15* allele also suppressed the slight developmental delay of the *lpo-6* (*mg360*) single-mutant strain (unpublished data). Finally, the *sgk-1* (*ft15*) mutation was inherited in a semi-dominant manner, and inactivation of *sgk-1* by RNAi abolished the ability of *sgk-1* (*ft15*) to suppress *lpo-6* (*mg360*) phenotypes (Figure 6C and 6D). Interestingly, *sgk-1* (*ft15*) is a weaker suppressor of the more severe, null *CeRictor* allele, *lpo-6* (*ft7*); in this background, it partially suppresses the small size phenotype and does not significantly reduce fat staining (Figure S4C and S4D). We do not currently know the biochemical consequences of the mutation caused by *ft15* on SGK-1 activity. Similarly, additional studies are needed to elucidate how SGK-1 activity is specifically affected by the partial or total loss of *CeRictor* function. Nevertheless, these genetic results strongly indicate that the *ft15* allele produces an activating mutation in SGK-1 and that this mutation suppresses nearly all of the phenotypes associated with *CeRictor*, demonstrating conclusively that *CeRictor* signals through *sgk-1* to regulate lipid storage, size, and development.

In sum, the observations that loss-of-function mutations in *sgk-1* mimic phenotypes of *CeRictor*, while a forward genetic screen aimed at finding suppressors of *CeRictor* identified an *sgk-1* gain-of-function mutation, indicate that *sgk-1* is a physiologically significant mediator of TORC2 activity in C. elegans.

## Discussion

We have found that loss of *Rictor* function causes developmental delay, reduced size and reproduction, but excess fat accumulation in C. elegans. Similar phenotypes have been observed by another group using independently generated alleles of *Rictor* (A. Soukas and G. Ruvkun, personal communication). As inactivation of another core TORC2 component, LST-8, recapitulates the *CeRictor* phenotypes, our findings suggest that, as in all other systems examined, *CeRictor* functions as part of the TORC2 complex. We found that the phenotypes caused by *CeRictor* inactivation were independent of activities of AKT and its downstream effector, the DAF-16/FOXO transcription factor. We further found that all of the phenotypes associated with loss of *CeRictor* were fully mimicked by loss of *sgk-1* and that these phenotypes were suppressed by a novel, gain-of-function mutation in *sgk-1.* These data indicate that *Rictor*, through TORC2, regulates fat storage, size, and development in C. elegans, and it governs all three phenotypes through upstream activation of *sgk-1*.

While the discovery that TORC2 phosphorylates AKT on its HM motif has been critical in characterization of various TORC2 complex components and identifying potential TORC2 targets based on HM domain homologies, the most prominent phenotypes caused by inactivation of TORC2 in C. elegans are independent of this activity. *CeRictor* appears to act through *sgk-1* but in parallel to both *Akt* genes to promote development, as the simultaneous loss of both *Akt* homologs and *CeRictor/sgk-1* results in a synergistic developmental delay. This genetic interaction occurs in the absence of *daf-16* activity, demonstrating FOXO-independent outputs for the *C. elegans Akt*s and *sgk-1*. Nevertheless, it is likely that, under certain circumstances, C. elegans TORC2 would modulate specific *Akt* functions. Indeed, the C. elegans AKT-1 protein does contain a conserved, PDK2 consensus phosphorylation site in its HM motif (unpublished data), suggesting that, as in all other organisms tested thus far, *C. elegans Akt* is likely a substrate of CeTORC2. In *Drosophila*, the only physiological role attributable to HM motif phosphorylation of AKT is modulation of insulin signaling specifically when the activity of the insulin pathway is abnormally enhanced, either by overexpression of FOXO transcription factor or inactivation of *PTEN*, a phosphatase that elevates PIP3 levels and thus enhances AKT activity [16]. Consistent with our findings, under normal growth conditions, abrogation of AKT's HM domain phosphorylation has minimal effects on AKT-dependent cell growth in *Drosophila* [16].

The TORC2-SGK regulatory pathway is conserved across phylogeny and is used for a variety of physiological processes. In S. cerevisiae, TORC2 regulates ceramide synthesis through activation of *YPK2* (an SGK homolog in S. cerevisiae), which in turn is required for membrane synthesis and active cell growth [24]. Gain-of-function mutations in *YPK2* suppress the lethality of TORC2 loss-of-function mutations [25], just as the *ft15* gain-of-function mutation in *sgk-1* suppresses the metabolic and growth phenotypes of *CeRictor*. Interestingly, in *Dictyostelium*, TORC2 is required to activate both the closest *Akt* and *Sgk* homologs, and both kinases regulate cell growth and chemotaxis [45]. In mammals, the regulation of SGK family kinases by mTORC2 and its physiological significance are just beginning to be probed. Recently, mTORC2 has been shown to be required for mSGK1 hydrophobic motif phosphorylation in mammalian cells, and immunoprecipitated mTORC2 directly phosphorylates mSGK1 in vitro [46]. Furthermore, phosphorylation of an SGK1-specific target is reduced in cells deficient in mTORC2 components [46]. Thus, SGK appears to be the predominant effector of TORC2 under normal growth conditions in S. cerevisiae and C. elegans, and a similar regulatory interaction is suggested by in vitro and cell-based studies in mammalian systems.

Whether TORC2 and SGK kinase family regulate fat metabolism in mammals is not yet known. A muscle-specific deficiency of *Rictor* has recently been reported, and *Rictor* seems to be largely dispensable for proper muscle cell morphology, function, and respiration [10]. While an adipose-specific knockout of *Raptor* has been described, a similar knockout of *Rictor* has not [11]. Whereas the C. elegans genome contains a single SGK gene, mammals have three. *Sgk1* and *Sgk3* knockout mice have surprisingly mild phenotypes, possibly due to redundant functions of SGKs. *Sgk2-*null mice have not yet been reported*. Sgk3-*null mice display a transient growth delay and defects in hair follicle development, whereas *Sgk1-*null mice are defective in salt balance under a salt-deficient diet and their glucose uptake by various tissues post glucose load is blunted [47,48]. The deep evolutionary origins of numerous mammalian and C. elegans fat regulatory pathways suggest that in mammals, as in C. elegans, mTORC2 and mSGK family of kinases may also play roles in lipid metabolism; likewise, it will be important to demonstrate whether mTORC2 activation is required for any of the processes known to be governed by the mSGKs, such as sodium transport and blood pressure regulation.

## Materials and Methods

### C. elegans strains and maintenance.

Nematodes were grown according to standard protocols at 20 °C [49]. N2 Bristol was used as wild-type strain. The following strains were used: *daf-16* (*mgDf47*) *I*, *daf-16* (*mgDf47*) *I;lpo-6* (*mg360*) *II*, *daf-16* (*mgDf47*) *I;lpo-6* (*mg360*) *II;akt-1* (*mg306*) *V;akt-2* (*tm812*) *X*, *daf-16* (*mgDf47*) *I;lpo-6* (*mg360*) *II;akt-1* (*mg306*) *V;akt-2* (*tm812*)*;sgk-1* (*ft15*) *X*, *daf-16* (*mgDf47*) *I;akt-1* (*mg306*) *V;akt-2* (*tm812*) *X*, *daf-16* (*mgDf47*) *I;akt-1* (*mg306*) *V;akt-2* (*tm812*)*;sgk-1* (*ok538*) *X*, *daf-16* (*mgDf47*) *I;sgk-1* (*ok538*) *X*, *sma-6* (*wk7*) *II*, *sma-6* (*wk7*) *II;lpo-6* (*mg360*) *II*, *lpo-6* (*mg360*) *II*, *lpo-6* (*mg360*) *II;lon-1* (*e185*) *III*, *lpo-6* (*mg360*) *II;egl-4* (*ad450sd*) *IV*, *lpo-6* (*m*,*g360*) *II;egl-4* (*n478*) *IV*, *lpo-6* (*mg360*) *II;dbl-1* (*nk3*) *V*, *lpo-6* (*ft7*) *II*, *lpo-6* (*mg360*) *II;akt-2* (*tm812*)*;sgk-1* (*ft15*) *X*, *lpo-6* (*ft7*) *II;akt-2* (*tm812*)*;sgk-1* (*ft15*) *X*, *lpo-6* (*ft7*)*;sgk-1* (*ok538*) *X*, *lpo-6* (*mg360*)*;ftEx560[P_lpo-6_::lpo-6genomic::polycis-GFP; Pmyo-2::mCherry]*, *lon-1* (*e185*) *III*, *egl-4* (*ad450sd*) *IV*, *egl-4* (*n478*) *IV*, *dbl-1* (*nk3*) *V*, *akt-2* (*tm812*)*;sgk-1* (*ft15*) *X*, *sgk-1* (*ok538*) *X*, *N2;ftEx645[P_lpo-6_::GFP; P_odr-1_::RFP]*.

### Lipid staining.

Nile Red staining and image analysis were performed as described previously [50,51]. Briefly, images were taken with identical exposure times and below saturation of pixel intensity. The first three pairs of anterior intestinal cells were selected (we previously demonstrated that this is generally representative of fat content quantification that includes the entire animal). Using ImageJ software, Nile Red–stained particles were enhanced by the SpotTracker Gaussian filter, a mask was created to remove background signal, and fluorescence intensity was measured. For all experiments, worms were synchronized by hypochlorite treatment of adults and imaged at the onset of egg-laying. All comparisons were conducted on animals that were at the same developmental stage. Due to the delay in developmental rate, strains containing *lpo-6* (*mg360*) were imaged 4–6-h later than wild type, strains containing *lpo-6* (*ft7*) or *sgk-1* were imaged 9–10 h later than wild type, and *lpo-6* (*mg360*)*;akt-1;akt-2;daf-16* worms were imaged ∼20 h later than wild type. Because of suppression of the developmental delay phenotype of *lpo-6* (*mg360*), the *lpo-6* (*mg360*)*;sgk-1* (*ft15*) was plated at the same time as *sgk-1* (*ft15*) and wild type. C. elegans were stained with BODIPY 500/510 C1,C12 in the same manner as that described for Nile Red, but the concentration was different: 1mg/ml BODIPY in DMSO stock was diluted 1:2,500 then 0.5 ml were added to 6-cm NGM plates seeded with OP50. Sudan Black staining was performed as described previously [50]. Representative images from single experiments are shown; each experiment was repeated on variably numerous occasions but at least twice. In each experiment 5–10 animals were quantified per genotype.

### Body size measurements.

Images of adult worms were taken at 5× magnification, and perimeter was traced and measured using Openlab software (Improvision). Pixel measurements were converted to microns by calibration using a stage micrometer. Animals were measured at the onset of egg-laying; developmental timing differences were adjusted as described above. Single experiments are shown—each experiment was performed at least twice, and in each experiment 10–20 animals were measured per genotype.

### RNAi.

RNAi by feeding was performed as described previously [52]. RNAi clone against *C10H11.8* (*lst-8* homolog) from the Ahringer feeding library was used [53]. RNAi was induced for two generations (synchronized adults were grown from L1s on HT115 expressing RNAi or L4440, then eggs harvested by hypochlorite treatment). Synchronized progeny were plated on RNAi plates with Nile Red and analyzed as adults. For *sgk-1* RNAi experiments, the RNAi clone against *W10G6.2* (*sgk-1*) from the Vidal feeding library was used [54]. In these experiments, RNAi was induced for one generation, starting as synchronized L1s.

### Rescue and promoter::GFP reporter constructs.

Entire genomic locus for *lpo-6* (F29C12.3) plus 1,039 base pairs of upstream sequence was amplified in three PCR fragments overlapping by 1 kb. The last fragment contained a polycistronic sequence fused to GFP and the *unc-54* 3′ UTR (gift of G. Brown) by PCR fusion. All three fragments were injected at 10 ng/μl into the gonad of *lpo-6* (*mg360*) adults. For expression pattern, rescuing promoter was amplified by PCR, cloned into Gateway vector (Invitrogen), and fused in-frame to GFP with the *unc-54* 3′ UTR. Plasmid was injected into N2 animals at 100 ng/μl with *P_odr-1_::RFP* as a coinjection marker.

### Feeding rate measurements.

Experiments were performed essentially as described previously [50]. Egg-laying adults were measured in each experiment; strains were adjusted for developmental differences as indicated above.

### Mutagenesis screening.

Ethyl methanesulfonate mutagenesis was performed as described previously [49]. Mutagenesis was carried out in two batches, totaling approximately 60,000 F_1_s and 300,000 F_2_s. For each batch, mutagenized *lpo-6* (*mg360*)*; akt-1; akt-2; daf-16* animals were allowed to grow to adulthood, and their progeny were collected by sodium hypochlorite treatment in eight separate pools. These were allowed to grow to adulthood, whereupon their progeny were collected in the same manner. Synchronized F_2_s were then allowed to grow for approximately 72 h at 20 °C (because of their developmental phenotype, *lpo-6* (*mg360*)*; akt-1; akt-2; daf-16* animals grown as described were an asynchronous mix of animals, none of which was older than very early L4 stage) then screened for the rare young or gravid adult animals. Potential suppressor lines were then retested on plates containing Nile Red, and only those whose developmental suppression was reproducible and further suppressed the increased staining of the original *lpo-6* (*mg360*)*; akt-1; akt-2; daf-16* strain were selected for future study.

## Supporting Information

Figure S1 Molecular Cloning of *CeRictor* Mutants (A) Gene structure for *lpo-6* (*F29C12.3*). Intron/exon boundaries are shown to scale. Below is a schematic of the LPO-6 protein; numbers reflect amino acid sequence of LPO-6 protein (there are no consensus domains in the protein as predicted by sequence homology). Sites and consequences of both *mg360* and *ft7* mutations are shown.
(B) Multiple-species alignment of conserved, C-terminal region where *mg360* allele substitution is found. Asterisk denotes the mutated residue.
(C) Expression pattern of rescuing *lpo-6* promoter fused to GFP in adult animal. Note exclusively intestinal expression pattern, with the exception of I1 interneuron. Image results from merge of fluorescence and DIC channels.
(D) Expression pattern of rescuing *lpo-6* promoter fused to GFP in early larval animal. Note both intestinal (I) and hypodermal (H) expression. Image results from merge of fluorescence and DIC channels.
(E) Expression of rescuing *lpo-6* promoter fused to GFP in I1 interneuron. Image results from merge of fluorescence and DIC channels. Neuron was identified as I1 due to the position of the cell body and a projection crossing the pharynx and extending anterior on the side opposite the cell body.
(3.26 MB PDF)

Figure S2 Increased-Fat Phenotype of *CeRictor* Mutants Is Confirmed by Other Lipid Staining Methods and Is Not Due to Developmental Timing (A) Images of fatty acid-conjugated BODIPY staining in adult wild type, *lpo-6* (*mg360*) and *lpo-6* (*ft7*) strains. Representative images are shown.
(B) Images of Sudan Black B staining in wild type, *lpo-6* (*mg360*) and *lpo-6* (*ft7*) strains. Sudan Black staining is generally less sensitive than Nile Red and Bodipy-conjugated fatty acids in detecting fat stores. Because of the variability of Sudan Black staining, we previously devised a protocol [50] allowing for pairwise comparison between strains stained within the same tube. Thus, representative images of wild-type and test animals from each experiment are shown.
(C) Comparison of fat accumulation as detected by Nile Red staining in *sgk-1* and *CeRictor* mutants. All strains were allowed to grow for the same amount of time after synchronized hatching by hypochlorite treatment of parents. Differences in developmental timing were not corrected. Mean fluorescence intensity is reported as a percentage of the mean for wild-type animals (error bars indicate s.e.m.). Asterisk indicates *p*-value <0.05 (wild type versus mutant, two-tailed *t*-test, *n* = 5–6).
(845 KB PDF)

Figure S3 Two-Generation *lst-8* RNAi Results in Fat and Size Phenotypes Similar to *CeRictor* Mutants (A) Representative images of control- and RNAi-treated animals (adult stage) stained with Nile Red. Note an increase in fluorescence intensity and an increase in lipid particle size, similar to what was noted with *lpo-6* mutants.
(B) Quantification of body size. RNAi value reported as mean size as a percentage of mean for control treatment (error bars indicate s.e.m.). Double asterisk indicates *p*-value <0.01 (control versus RNAi, two-tailed *t*-test, *n* = 10). Note that body size of RNAi-treated animals is nearly identical to that of *lpo-6* (*ft7*) mutants (see also Figure 1D).
(714 KB PDF)

Figure S4 Molecular Cloning of *sgk-1* (*ft15*) and Epistasis Analyses with *lpo-6* (*ft7*) (A) Schematic representation of CeSGK-1, showing to scale its conserved domains, putative phosphorylation sites (TM = turn motif and HM = hydrophobic motif), and location and consequence of *ft15* mutation.
(B) Multiple-species alignment of conserved region where *ft15* allele substitution is found. Asterisk denotes the mutated residue.
(C) Quantification of body size. Values reported as mean size as a percentage of mean for wild-type animals (error bars indicate s.e.m.). Double asterisk indicates *p*-value <0.01 (wild type versus mutant strain or *lpo-6* (*ft7*) vs. *lpo-6* (*ft7*)*;sgk-1* (*gf*), two-tailed *t*-test, *n* = 10). In all panels, *sgk-1* (*gf*) refers to the *sgk-1* (*ft15*) allele.
(D) Quantification of Nile Red staining. Mean fluorescence intensity is reported as a percentage of the mean for wild-type animals (error bars indicate s.e.m.). Double asterisk indicates *p*-value <0.01 (wild type versus mutant, two-tailed *t*-test, *n* = 5–8). *lpo-6* (*ft7*)*;sgk-1* (*gf*) does not differ significantly from *lpo-6* (*ft7*) or *sgk-1* (*gf*) (*p*-values = 0.39 and 0.07, respectively).
(817 KB PDF)

## Abbreviations

HM: hydrophobic motif
Rictor: rapamycin-insensitive companion of mTOR
TOR: target of rapamycin
