# Supplementary material for: Rictor/TORC2 Regulates Caenorhabditis elegans Fat Storage, Body Size, and Development through sgk-1
Source: PLoS Biol. 2009 Mar 3;7(3):e1000060. doi: 10.1371/journal.pbio.1000060 (PMC2650726; doi:10.1371/journal.pbio.1000060)
Supplement: Supplementary file 1 [file pbio.1000060.sg001.pdf]

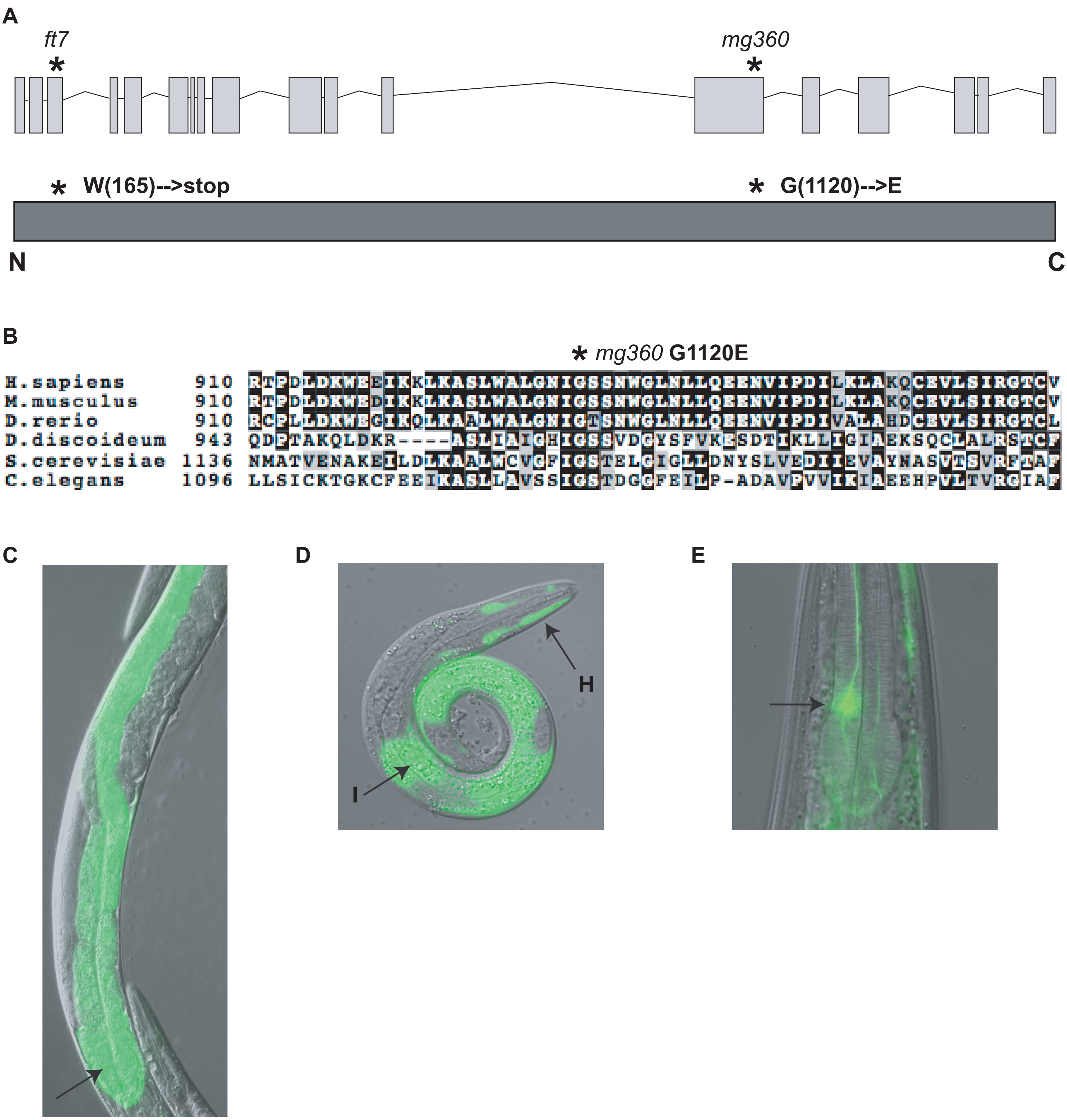
A
ft7
mg360
* W(165)-->stop
* G(1120)-->E
N
C
B
* mg360 G1120E
H.sapiens 910 RTPDLDKWEEIKKLKASLWALGNIGSSNWGLNLLQEENVIPDILKLAKQCEVLSIRGTCV
M.musculus 910 RTPDLDKWEDIKKLKASLWALGNIGSSNWGLNLLQEENVIPDILKLAKQCEVLSIRGTCV
D.rerio 910 RCPLLDKWEGIKQLKAALWALGNIGTSNWGLNLLQEENVIPDIVALAHDCEVLSIRGTCL
D.discoideum 943 QDPTAKQLDKR----ASLIAIGHIGSSVDGYSFVKESDTIKLLIGIAEKSQCLALRSTCF
S.cerevisiae 1136 NMATVENAKEILDLKAALWCVGFIGSTELGIGLLDNYSLVEDIIEVAYNASVTSVRFTAF
C.elegans 1096 LLSICKTGKCFEEIKASLLAVSSIGSTDGGFEILP-ADAVFVVIKIAEEHPVLTVRGIAF
C
D
H
I
E
