# Supplementary material for: Rictor/TORC2 Regulates Caenorhabditis elegans Fat Storage, Body Size, and Development through sgk-1
Source: PLoS Biol. 2009 Mar 3;7(3):e1000060. doi: 10.1371/journal.pbio.1000060 (PMC2650726; doi:10.1371/journal.pbio.1000060)
Supplement: Supplementary file 2 [file pbio.1000060.sg002.pdf]

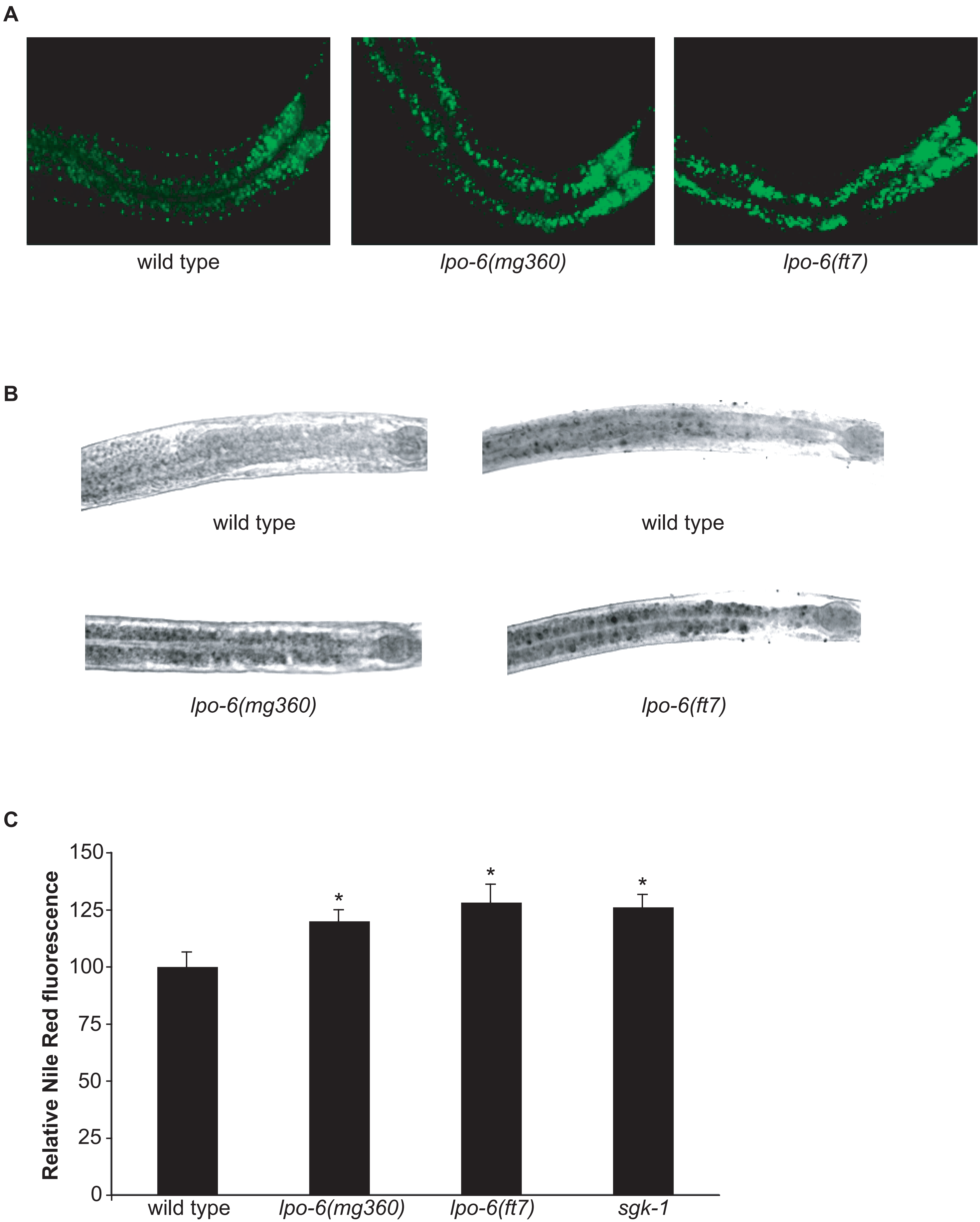
A
wild type
lpo-6(mg360)
lpo-6(ft7)
B
wild type
wild type
lpo-6(mg360)
lpo-6(ft7)
C
Relative Nile Red fluorescence
150
125
100
75
50
25
0
*
*
*
wild type
lpo-6(mg360)
lpo-6(ft7)
sgk-1
