# Supplementary material for: Rictor/TORC2 Regulates Caenorhabditis elegans Fat Storage, Body Size, and Development through sgk-1
Source: PLoS Biol. 2009 Mar 3;7(3):e1000060. doi: 10.1371/journal.pbio.1000060 (PMC2650726; doi:10.1371/journal.pbio.1000060)
Supplement: Supplementary file 3 [file pbio.1000060.sg003.pdf]

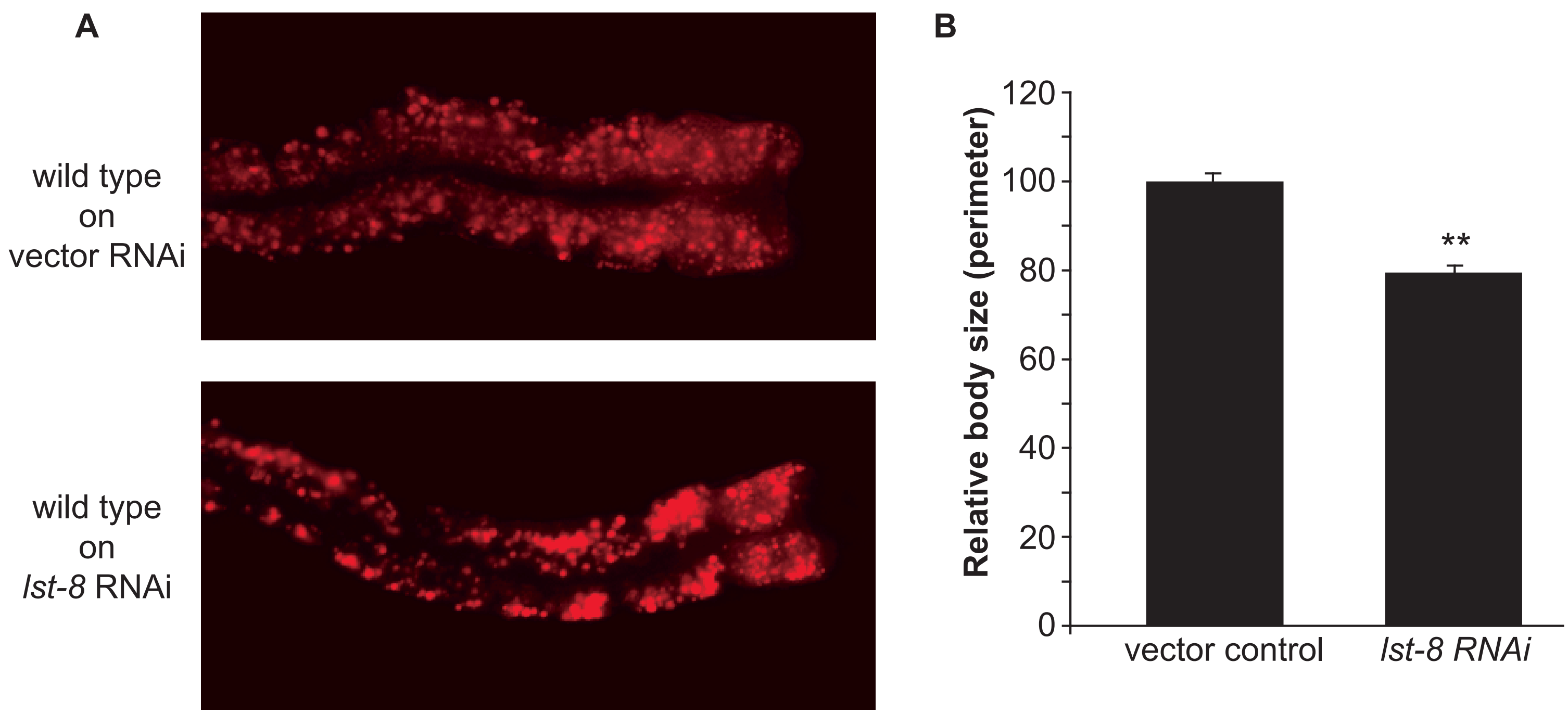
A
wild type
on
vector RNAi
wild type
on
lst-8 RNAi
B
Relative body size (perimeter)
120
100
80
60
40
20
0
**
vector control
lst-8 RNAi
