# Supplementary material for: Rictor/TORC2 Regulates Caenorhabditis elegans Fat Storage, Body Size, and Development through sgk-1
Source: PLoS Biol. 2009 Mar 3;7(3):e1000060. doi: 10.1371/journal.pbio.1000060 (PMC2650726; doi:10.1371/journal.pbio.1000060)
Supplement: Supplementary file 4 [file pbio.1000060.sg004.pdf]

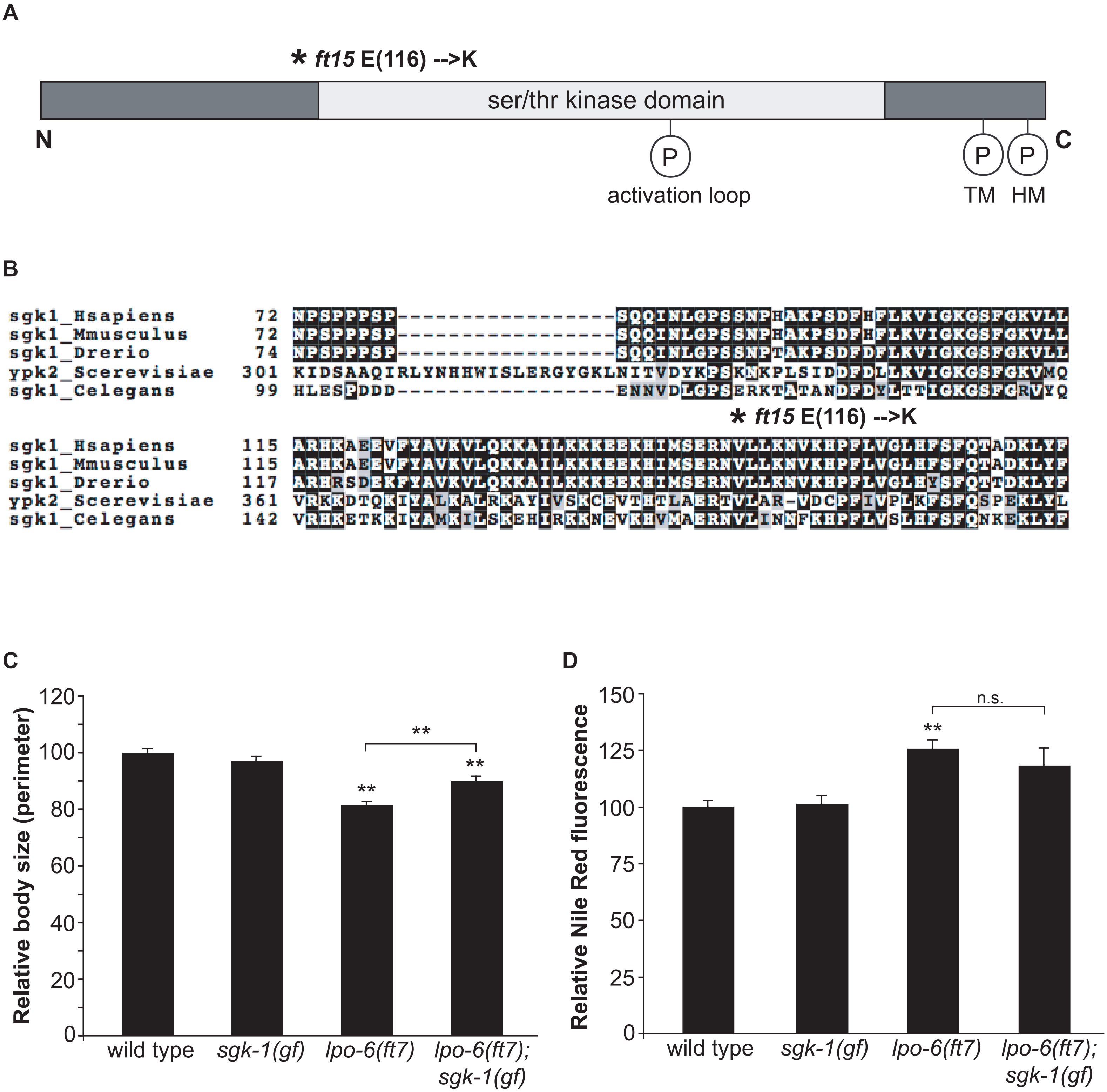
A
* ft15 E(116) -->K
ser/thr kinase domain
N
C
P
activation loop
P
P
TM
HM
B
sgk1_Hsapiens 72 NPSPPPSP-----------------SQQINLGPSSNPHAKPSDFHFLKVIGKGSFGKVLL
sgk1_Mmusculus 72 NPSPPPSP-----------------SQQINLGPSSNPHAKPSDFHFLKVIGKGSFGKVLL
sgk1_Drerio 74 NPSPPPSP-----------------SQQINLGPSSNPTAKPSDFDFLKVIGKGSFGKVLL
ypk2_Scerevisiae 301 KIDSAAQIRLYNHHWISLERGYGKLNITVDYKPSKNKPLSIDDFDLLKVIGKGSFGKVMQ
sgk1_Celegans 99 HLESPDDD-----------------ENNVDLGPSERKTATANDFDYLTTIGKGSFGRVYQ
* ft15 E(116) -->K
sgk1_Hsapiens 115 ARHKAEEVFYAVKVLQKKAILKKKEEKHIMSERNVLLKNVKHPFLVGLHFSFQTADKLYF
sgk1_Mmusculus 115 ARHKAEEVFYAVKVLQKKAILKKKEEKHIMSERNVLLKNVKHPFLVGLHFSFQTADKLYF
sgk1_Drerio 117 ARHRSDEKFYAVKVLQKKAILKKKEEKHIMSERNVLLKNVKHPFLVGLHYSFQTTDKLYF
ypk2_Scerevisiae 361 VRKKDTQKIYALKALRKAYIVSKCEVTHTLAERTVLAR-VDCPFIVPLKFSFQSPEKLYL
sgk1_Celegans 142 VRHKETKKIYAMKILSKEHIRKKNEVKHVMAERNVLINNFKHPFLVSLHFSFQNKEKLYF
C
Relative body size (perimeter)
0
20
40
60
80
100
120
**
**
**
wild type
sgk-1(gf)
lpo-6(ft7)
lpo-6(ft7); sgk-1(gf)
D
Relative Nile Red fluorescence
0
25
50
75
100
125
150
n.s.
**
wild type
sgk-1(gf)
lpo-6(ft7)
lpo-6(ft7); sgk-1(gf)
